# Dexamethasone suppresses immune evasion by inducing GR/STAT3 mediated downregulation of PD-L1 and IDO1 pathways

**DOI:** 10.1038/s41388-021-01897-0

**Published:** 2021-06-26

**Authors:** Zhen Xiang, Zhijun Zhou, Shuzheng Song, Jun Li, Jun Ji, Ranlin Yan, Jiexuan Wang, Wei Cai, Wenjun Hu, Lu Zang, Zhenggang Zhu, Zhen Zhang, Min Li, Yingyan Yu

**Affiliations:** 1 Department of General Surgery of Ruijin Hospital, Shanghai Institute of Digestive Surgery, and Shanghai Key Laboratory for Gastric Neoplasms, Shanghai Jiao Tong University School of Medicine, Shanghai, China; 2 Department of Medicine, The University of Oklahoma Health Sciences Center, Oklahoma City, OK 73104 USA; 3 Department of Surgery, The University of Oklahoma Health Sciences Center, Oklahoma City, OK 73104 USA; 4 Department of Radiation Oncology and Department of Oncology, Shanghai Medical College, Fudan University Shanghai Cancer Center, Shanghai, PR China, 270 Dong An Road, Shanghai, 200032 China

**Keywords:** Gastrointestinal cancer, Immunotherapy

## Abstract

T cell exhaustion plays critical roles in tumor immune evasion. Novel strategies to suppress immune evasion are in urgent need. We aimed to identify potential compounds to target T cell exhaustion and increase response to immune checkpoint inhibitors (ICIs). Differentially expressed genes (DEGs) were identified between tumors with different immune evasion potential by comparing the transcriptome data. DEGs were then analyzed in the Connectivity Map (CMap) platform to identify potential compounds to increase response to ICIs. Gene set enrichment analysis, LDH release assay, Chromatin immunoprecipitation (ChIP), and Co-IP were performed to explore the potential mechanisms in vitro. Patients derived organoids and humanized xenograft mouse model were utilized to validate the finding ex vivo and in vivo. We identified 25 potential compounds that may play critical roles in regulating tumor immune evasion. We further pinpointed a specific compound, dexamethasone, which shows potent anti-tumor effect in multiple cancer cell lines when cocultured with T cells. Dexamethasone can suppress T cell exhaustion by decreasing the activity of two immune checkpoints simultaneously, including PD-L1 and IDO1. Functional study shows dexamethasone can increase the sensitivity of ICIs in coculture system, 3D organoid model and humanized mouse model. Mechanism study shows dexamethasone mediated transcriptional suppression of PD-L1 and IDO1 depends on the nuclear translocation of GR/STAT3 complex. These findings demonstrate dexamethasone can suppress immune evasion by inducing GR/STAT3 mediated downregulation of PD-L1 and IDO1 pathways.

## Introduction

T cell immunity against cancers is regulated by co-stimulatory signals and co-suppressive signals. The co-suppressive signals are called immune checkpoints, which weaken T cell functions and play critical roles in tumor immune escape [1]. The immune inhibitory molecules of cancer cells include BTLA, VISTA, CD160, PD-L1, CTLA4, IDO1, LAG3, LGALS9, TNFRSF14, VTCN1 and so on [2–9]. Several US FDA approved immune checkpoint inhibitors (ICI), including Ipilimumab (anti-CTLA4), Pembrolizumab and Nivolumab (anti-PD1/PD-L1) have impressive anti-tumor effect in a subset of tumors [10]. Combination of Nivolumab and Ipilimumab significantly prolonged progression-free survival compared with Ipilimumab or Nivolumab alone in unresectable melanoma patients [11]. Wang et al. found that LAG-3 and its novel ligand fibrin 1 (FGL1)/LAG-3 functions as new immune checkpoint pathway independent of the PD-1/PD-L1 pathway, and may be new target of cancer resistant to anti-PD1 treatment [12]. Therefore, targeting multiple immune checkpoints may be a promising strategy to overcome immune checkpoint inhibitors and acquired more anti-tumor effects in cancers [1].

Cancer Cell Line Encyclopedia (CCLE) is an open database consisting of genomic, transcriptomic and methylation data of over 1000 cancer cell lines covering 24 types of cancer [13]. The Cancer Genome Atlas (TCGA) database consists of multi-omics data of more than 11,000 cases from 33 types of cancer [14]. Connectivity Map (CMap) database is an open database consisting of transcriptomic data pre- and post drugs treatment of over 2000 small compounds on nine cancer cell lines, and often used for drug reposition study [15, 16]. Scott et al. found drugs targeting MEK and TOP2A may be highly efficacious against metastatic pancreatic neuroendocrine tumors by CMap analysis and in vitro cytotoxicity assays [17]. Recently, we also used CMap database to identify the glucocorticoids as the potential drugs to upregulate ACE2 expression, such as dexamethasone and hydrocortisone, which indicated glucocorticoids may be a drug to treat COVID-19 patients [18]. In a randomized, controlled clinical trial, Ledford et al. found that dexamethasone could reduce death rates of COVID-19 patients [19].

In the current study, we carried out cross-database analysis on multiple immune-related molecules and potential targeted therapies. A total of 25 potential compounds were identified, which may play critical roles in regulating tumor immune evasion. Among them, dexamethasone shows potent anti-tumor effect in multiple cancer cell lines when cocultured with T cells. Dexamethasone can decrease T cell exhaustion by suppressing the activity of two immune checkpoints simultaneously, including PD-L1 and IDO1. Functional study shows dexamethasone can increase the sensitivity of ICIs in coculture system, 3D organoid model and humanized mouse model. Mechanism study shows dexamethasone mediated transcriptional suppression of PD-L1 and IDO1 depends on the nuclear translocation of GR/STAT3 complex. These findings suggest dexamethasone as a potential regimen to increase sensitivity of ICIs.

## Materials and methods

### Cross-database analysis

Transcriptomic data was obtained from CCLE (https://portals.broadinstitute.org/ccle) and TCGA (The Cancer Genome Atlas, RRID:SCR_003193). The mRNA levels of 10 critical regulators of tumor immune evasion were manually curated, including BTLA, VISTA, CD160, PD-L1, CTLA4, IDO1, LAG3, LGALS9, TNFRSF14, and VTCN1. Then we performed correlation analysis between these regulators. Those with correlation coefficients larger than 0.3 or less than −0.3 were presented by heatmap using “pheatmap” package in R. The CMap database (RRID:SCR_016204) was used for matching chemicals based on 150 differential expressed genes between PD-L1(+)/IDO1(+) vs PD-L1(−) /IDO1(−) GC or CRC cancer cells.

### Cell lines and reagents

Cell lines were obtained from American Type Culture Collection, Japanese Collection of Research Bioresources Cell Bank and several other cell banks (Supplementary Table S1). Cell lines were authenticated using short tandem repeat (STR) profiling and were tested negative for mycoplasma contamination. Cancer cell lines were cultured in RPMI-1640 containing 10% FBS and 1% penicillin–streptomycin, and incubated at 37 °C in a humidified incubator at 5% CO_2_. Dexamethasone (S1322, Houston, USA) and Pembrolizumab (A2005, Houston, USA) were purchased from SELLECK. Other chemicals and antibodies were listed in Supplementary Table S1. Eukaryotic expressing plasmids of pcDNA3.1-pGFP-PD-L1 or pcDNA3.1-pGFP-IDO1 were purchased from GeneChem (Shanghai). Plasmid transfection was performed using Lipofectamine 2000 (Invitrogen, Carlsbad, California, USA).

### 3D organoid model

GC tissues were obtained from Department of Surgery, Ruijin Hospital of Shanghai Jiaotong University School of Medicine. No chemotherapy, radiotherapy, or immunotherapy was received pre-surgery. This study was approved by the Research Ethics Committee of Shanghai Ruijin hospital. The written informed consents were signed by all patients. A 1 cm^3^ fresh tissue was cut and soaked in PBS containing 5% penicillin–streptomycin and antibiotics (2% primocin, Invitrogen, California, USA) for 30 min, and then, minced for enzymatic digestion by 1.5 mg/mL collagenase IV (7426, STEMCELL, British Columbia, Canada) for 1 h at 37 °C. Cells were washed twice by advanced DMEM/F12, and filtered by 40 µm cell strainer (27305, STEMCELL, British Columbia, Canada). After centrifuged for 5 min at 1000 rpm, cells were seeded into Matrigel (356235, Corning, New York, USA) and overlayed with human IntestiCult™ organoid growth medium (06010, STEMCELL, British Columbia, Canada) containing 10 µM Y-27632, 1% Primocin and 1% penicillin–streptomycin. TrypLE™ Express Enzyme (12604021, Thermo Scientific, Massachusetts, USA) was used to digest and resuspended cancer cells for organoid passages.

### Co-immunoprecipitation (Co-IP) assay

After incubation with 50 nM dexamethasone for 48 h, cancer cells (2 × 10^6^) were washed with PBS, and harvested with immunoprecipitation lysis buffer (P0013, Beyotime, Beijing). Antibodies to STAT3, GR, and magnetic beads (20 μL, 88802, Thermo Scientific, Massachusetts, USA) were added to lysates and incubated for 1 h at 4 °C. After washing three times with immunoprecipitation lysis buffer, the precipitates were analyzed with antibody against STAT3 or GR. The working dilutions of antibodies were stated in Supplementary Table S1.

### Chromatin immunoprecipitation (ChIP) assay

ChIP Assay Kit (17-371, Millipore, Massachusetts, USA) was used. Briefly, cancer cells (5 × 10^6^) were cross-linked with formaldehyde at a final concentration of 1% for 10 min at room temperature, then stopped by glycine. Chromatin was sonicated in the lysis buffer to 100–300 bp. One-tenth of the total lysate was removed to use as an input DNA control. Supernatant fraction was pre-cleared with 50% slurry of Salmon Sperm DNA/Protein A/G agarose for 1 h at 4 °C on a rocker. Immunoprecipitation was performed with anti-STAT3 and control rabbit IgG (Supplementary Table S1). Protein A/G agarose was applied to pull down the target protein. The protein was digested with proteinase K, and the immunoprecipitated DNA was harvested and analyzed by PCR. Specific primers for promoters of PD-L1 and IDO1 are listed in Supplementary Table S1.

### Western blot and qPCR

Western blot and qPCR were performed as previously described [20]. The working dilutions of antibodies are listed in Supplementary Table S1. TRIzol solution (15596-026, Invitrogen, California, USA) and reverse transcription kit (FSQ-101, TOYOBO, Osaka, Japan) were used to examine mRNA level. SYBR™ Select Master Mix (4472908, Applied Biosystems, Foster City, USA) was used for qPCR in 10 μl reaction mixtures in HT 7900 (Applied Biosystems, Foster City, USA). The sequences of primers are listed in Supplementary Table S1.

### RNA-seq and gene set enrichment analysis

After incubation with 50 nM dexamethasone for 24 h, the total RNA was extracted for RNA-seq. The TruSeq RNA Sample Preparation kit (Illumina, San Diego, CA, USA) was used for library construction. Illumina HiSeq Xten (Illumina, San Diego, CA, USA) was used for RNA-seq. Gene counts were normalized to transcripts per million (TPM). Gene Set Enrichment Analysis (GSEA) was performed for pathway analysis by “clusterProfiler” package in R (clusterProfiler, RRID:SCR_016884). The top 20 pathways were displayed by bubble diagram utilizing “ggplot2” package in R (ggplot2, RRID:SCR_014601).

### LDH release assay

The lactate dehydrogenase (LDH) releasing was evaluated as cell damage by LDH Assay Kit-WST CK12 (CK12, DOJINDO, Kumamoto, Japan). Injury rate (%) of cancer cells was calculated as: (experimental LDH release-spontaneous LDH release)/(total LDH release-spontaneous LDH release).

### Cell proliferation assay and apoptosis detection

Cell Counting Kit-8 kit was purchased from DOJINDO (CK04, Kumamoto, Japan). Cancer cells (5000) or PBMCs (50,000) were placed on 96-well plates (100 μl/well). The experiments were performed as previously described [21]. After co-culture of cancer cells with PBMCs for 48 h, cells were harvested and stained with the antibodies labeled by fluorescence for 30 min in 4 °C in darks. Propidium Iodide (PI) (556463, BD, New Jersey, USA), FITC Annexin V (556420, BD, New Jersey, USA) were used. Fluorescence intensity was assayed by FACS Ariar (BD, New Jersey, USA), and analyzed using FlowJo 7.6.1 software (FlowJo, RRID:SCR_008520).

### Kynurenine assay

The kynurenine was analyzed by ultra-performance liquid chromatography mass spectrometry (UPLC/MS). The cell medium of 300 μl was mixed with 600 μl acetonitrile for 10 min rotation, and centrifuged at 12000 rpm for 10 min. A 1 μl supernatant was injected into a 2.1 × 100 mm column of ACQUITY UPLC BEH Amide. The mobile phase contained 0.1% formic acid (solvent A) and acetonitrile (solvent B) at a flow rate of 0.3 ml/min at 40 °C. Chromatography was performed as following: 5% A, held for 1 min; 1–3 min, increased to 50% A; 3–5 min, held at 50% A; 5–5.1 min, decreased to 5% A; 5.1–7 min, held at 5% A. Mass spectrometry was performed in the conditions of source temperature 400 °C, capillary voltage 1.5 kV, cone voltage 10 V. The Cone Gas Flow was 10 L/Hr, and the Desolvation Gas Flow was 800 L/Hr. High-purity helium and nitrogen were used for the collision gas. The Masslynx mass spectrometry data acquisition software (Waters Corp., Milford, USA) was used for extracting m/z, retention time and ion intensity. All spectra were aligned and normalized to the total peak intensity.

### Isolation and activation of human PBMCs

Peripheral blood was got from a healthy donor who has signed informed consent. Peripheral blood mononuclear cells (PBMCs) were separated using FICOLL PAQUE PLUS (17144003-1, GE, Buckinghamshire, England). Non-adherent cells were harvested and suspended in AIM-V medium supplemented with 1000 U/ml IFN-γ on day 1. Then, human CD3 NALE HIT3a (100 ng/ml) and human recombinant IL-2 (300 U/ml) were added at day 2 and day 3. The cell culture medium was half replaced by AIM-V medium containing 10% FBS, IL-2 (200 U/ml), IL-7 (25 ng/mL), and IL-15 (10 ng/mL) every 2-day.

### Immune reconstituted NOD/SCID model

Female NOD/SCID mice (4-week-old) were used (Shanghai Lingchang Biotechnology, Co. LTD, Shanghai). The animal experiments were approved by the Research Ethics Committee of Shanghai Jiaotong University School of Medicine. Simple randomization was applied to allocate the mice into different groups (*n* = 5 per group) according to literature and our previous studies. Investigators were blinded to the group allocation. In this study, animal experiment was performed on mouse model with reconstituted immune system [22], in which, NOD/SCID mice were infused PBMCs (2 × 10^7^) via tail veins at 1st and 14th day. One day after PBMCs infusion, 100 µL mixture of SGC-7901 or SW1990 (5 × 10^6^) and Matrigel was inoculated subcutaneously. Dexamethasone (0.1 mg/kg, 0.5 mg/kg, 5 mg/kg) was injected intraperitoneal every three days, and pembrolizumab (5 mg/kg) was injected via tail vein every week. The same experiments of SGC-7901 were also performed in non-immune reconstituted NOD/SCID model, which was not injected human PBMCs to humanize the immune system.

### Immunohistochemistry and immunofluorescence

Xenografts were fixed with 10% formalin, embedded with paraffin. Tissue sections were cut into 4-µm thick and stained for hematoxylin-eosin (HE) staining and immunohistochemistry. Cancer organoids were embedded in Optimum Cutting Temperature Compound (OCT), and cut into 6-µm thick for hematoxylin–eosin (HE) and protein expression analysis [20]. The working dilutions of antibodies were stated in Supplementary Table S1. To quantify the expression of PD-L1, IDO1, STAT3, GR, GZMA, PRF-1, Caspase3, and Caspase8, we referenced the methods in our previous study [20]. For scoring of CD8, we randomly selected five high power fields (HPF, 400×) and counted the positive cells in every fields. Finally, the average value of five fields was calculated. Blinding strategy was carried out during the evaluation of the slides by two independent pathologists.

### Statistics

All results are expressed as mean ± 95% CI from three repeats. Pearson correlation analysis of PD-L1 and IDO1 was performed. The student’s *t* test was carried out in GraphPad Prism 6.0 (GraphPad Prism, RRID:SCR_002798) unless otherwise specified. The variance is similar between the groups that are being statistically compared. Correlation diagrams were plotted by hierarchical clustering by “corrplot” package in R software. Gene ontology (GO) analysis was performed using online tool Metascape (Metascape, RRID:SCR_016620). Alpha was set to 0.05 in the power analysis. *P* value less than 0.05 was considered significant. RNA-seq data of cancer cells treated by dexamethasone was uploaded to the GEO repository (GSE134195).

## Results

### Discovery of drugs inhibiting PD-L1 and IDO1 of cancer cells simultaneously

**Fig. 1:**
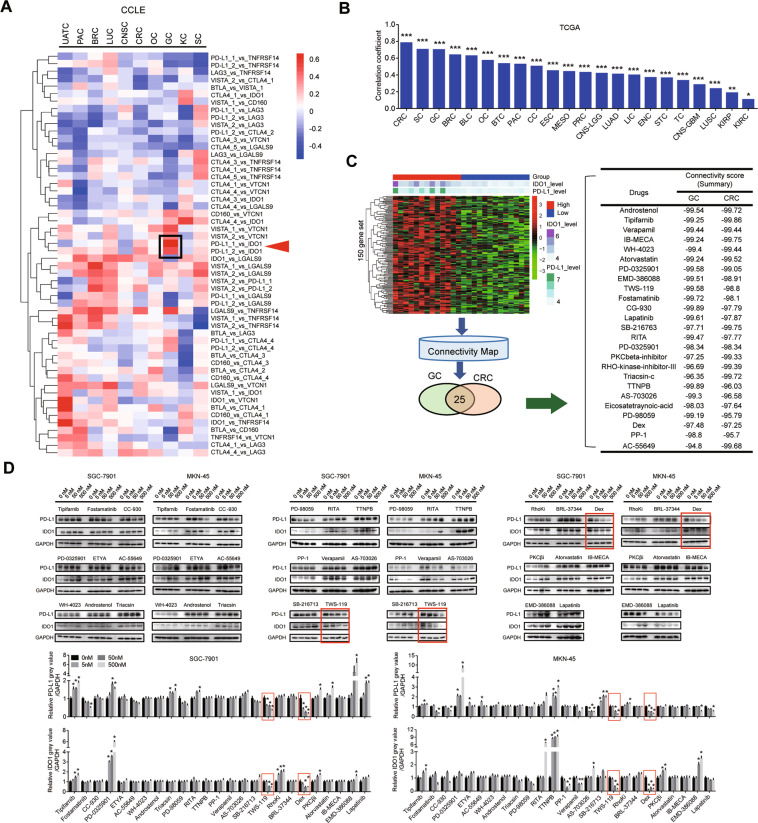
Potential compounds to target PD-L1 and IDO1 simultaneously. **A** The heatmap of correlation coefficients over 0.3 was plotted in heatmap. The probe numbers were listed on right side. The close correlation of PD-L1 and IDO1 was indicated on the heatmap. UATC upper aerodigestive tract cancer, PAC pancreatic cancer, BRC breast cancer, LUC lung cancer, CNS central nervous system, CRC colorectal cancer, OC Ovarian cancer, GC gastric cancer, KC kidney cancer, SC skin cancer. **B** The overview of correlation coefficient between PD-L1 and IDO1 expression across 20 types of cancers in TCGA. BTC biliary tract cancer, ESC esophageal cancer, LIC liver cancer, STC soft tissue cancer, ENC endometrial cancer, TC thyroid cancer, PRC prostate cancer, MESO mesothelioma, BLC bladder cancer, CC cervical cancer, CNS-LGG lower grade glioma, CNS-GBM glioblastoma multiform, LUAD lung adenocarcinoma, LUSC lung squamous cell carcinoma, KIRP kidney renal papillary cell carcinoma, KIRC kidney renal clear cell carcinoma. *P < 0.05; **P < 0.01; ***P < 0.001. **C** The differentially expressed gene sets between PD-L1(+)/IDO1(+) vs PD-L1(−)/IDO1(−) of GC and CRC were imported into CMap (left). The potential sensitive chemicals were exported (right). Dex Dexamethasone, RhoKi RHO-kinase-inhibitor-III, PKCβi PKCβ-inhibitor, ETYA Eicosatetraynoic-acid. **D** Cancer cell lines co-expressed PD-L1 and IDO1 (SGC-7901 and MKN-45) were used for efficacy screening of compounds at different concentrations (5 nM; 50 nM and 500 nM) for 48 h. Expression of PD-L1 and IDO1 was detected by Western blot. The red frame indicated the obvious efficacy of some compounds on suppressing PD-L1 and IDO1. *P < 0.01.

We performed correlation analysis of transcriptomic data of immune-related molecules BTLA, VISTA, CD160, PD-L1, IDO1, CTLA4, LAG3, LGALS9, TNFRSF14, and VTCN1 on multiple cancer cell lines in CCLE database. PD-L1 and IDO1 show the strongest correlation, especially in gastrointestinal cancers. The coefficient of two probes for PD-L1 were 0.675 and 0.502 in GC, (*P* < 0.05), and were 0.224 and 0.354 in CRC (*P* = 0.08 and *P* < 0.05, respectively) (Fig. 1A, Supplementary Fig. S1A). We further validated these findings in pan-cancer analysis in TCGA database and found the correlation coefficients of PD-L1 and IDO1 in GC and CRC patients’ tissues were 0.701 (*P* < 0.001) and 0.783 (*P* < 0.001), respectively (Fig. 1B).

Since the co-expression of PD-L1 and IDO1 was strongest in GC (correlation coefficient is 0.675) and CRC (correlation coefficient is 0.354), we further analyzed the transcriptomic data of 38 GC cell lines and 61 CRC cell lines in CCLE database. We set median value of PD-L1 or IDO1 as cutoff value, and stratified them into four groups: PD-L1(−)/IDO1(−) (GC: *n* = 13, CRC: *n* = 19), PD-L1(−)/IDO1(+) (GC: *n* = 6, CRC: *n* = 11), PD-L1(+)/IDO1(−) (GC: *n* = 6, CRC: *n* = 12) and PD-L1(+)/IDO1(+) (GC: *n* = 13, CRC: *n* = 19). We collected the top 150 upregulated genes in PD-L1(+)/IDO1(+) tumors compared to PD-L1(−)/IDO1(−) tumors, and applied them for CMap analysis. A series of connective compounds mimicking the phenotypes were identified. According to the connectivity scores, we selected compounds negatively regulating PD-L1 and IDO1 in GC and CRC (connectivity score <−90), and identified top 25 compounds as potential inhibitors for PD-L1 and IDO1 (Fig. 1C and Supplementary Table S2). By evaluating inhibitory efficacy of 25 candidate compounds (0–500 nM) in cancer cell lines (SGC-7901 and MKN45 cells), dexamethasone and TWS-119 revealed obvious inhibitory effect on PD-L1 and IDO1 in a dose-dependent manner (Fig. 1D, Supplementary Fig. S1B).

### Dexamethasone decreases T cell exhaustion

**Fig. 2:**
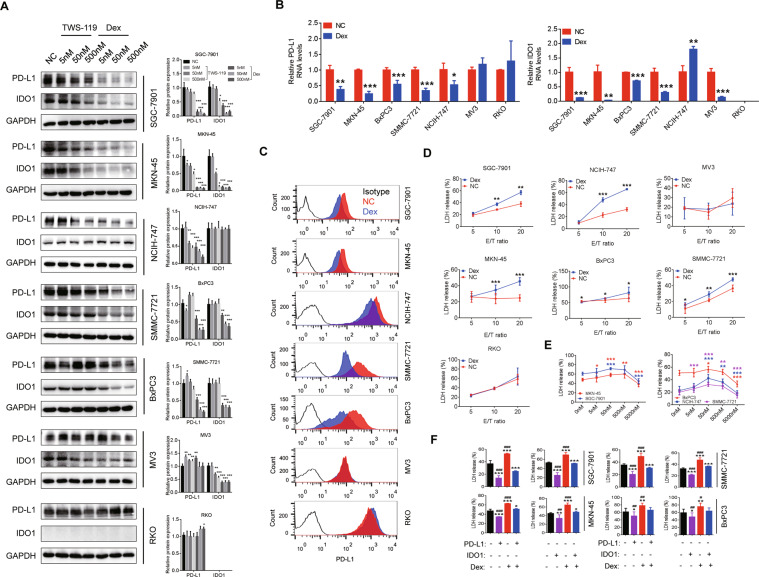
Low-dose dexamethasone enhances anti-tumor immunity by suppressing PD-L1 and IDO1 expression in vitro. **A** Expression of PD-L1 and IDO1 on cancer cell lines after treated with different doses of dexamethasone or TWS-119 (50 nM) for 48 h; (**B**) mRNA levels of PD-L1 and IDO1 on cancer cell lines after treated with different doses of dexamethasone (50 nM) for 48 h; (**C**) Expression of PD-L1 after dexamethasone (50 nM) treatment for 48 h was detected by flow cytometry analysis. **D** The LDH release of cancer-PBMCs co-cultivation system with effector/target ratio of 5:1, 10:1, and 20:1 was examined. These experiments were repeated three times. **E** Cytotoxicity of PBMCs against cancer cells was measured. Pre-treated cancer cells and PBMCs with different concentrations of dexamethasone for 48 h; and then co-cultured for another 48 h; LDH release and cytotoxicity against cancer cells was detected. **F** Enforcing PD-L1 or IDO1 expression in cancer cells and then evaluated the cytotoxicity against tumor cells in cancer-PBMCs co-cultivation system with effector/target ratio of 10:1. “*” indicates comparison with NC group; “#” indicates comparison with “dexamethasone and PD-L1” or “dexamethasone and IDO1”. “Dex” indicates “dexamethasone”, and “NC” indicates “Negative Control”. *P < 0.05; **P < 0.01; ***P < 0.001. #*P* < 0.05; ##*P* < 0.01; ###*P* < 0.001.

We treated cancer cells with dexamethasone and TWS-119, and found that dexamethasone can suppress PD-L1 and IDO1 expression in multiple cancer cell lines, including SGC-7901, MKN-45, SMMC-7721, and BxPC3 (Fig. 2A–2B and Supplementary Fig. S1B). Interestingly, dexamethasone showed stronger suppressive effects than TWS-119 did. Given that dexamethasone is an FDA certified anti-inflammatory drug widely used in clinic, we focused on dexamethasone in the next experiments. We further confirmed the decreased expression of PD-L1 on cell membrane by flow cytometry (FCM) analysis (Fig. 2C). Considering IDO1 is a rate-limiting enzyme for catalyzing tryptophan to kynurenine, we detected the level of kynurenine, and found it decreased in culture medium of SGC-7901, MKN-45, BxPC3, and SMMC-7721 cell lines (Supplementary Fig. S2A) after treatment with dexamethasone for 48 h. We observed anti-proliferative effects of dexamethasone in SGC-7901, MKN-45, SMMC-7721, and NCIH-747 and MV3 cell lines (Supplementary Fig. S2B). In PBMCs-cancer co-cultivation system, cytotoxicity of T cells can be evaluated by the level of lactate dehydrogenase (LDH) released by cancer cells. LDH release assay showed that dexamethasone treatment could not directly induce the LDH release in cancer cells when they were not co-cultured with PBMCs (Supplementary Fig. S2C). Interestingly, it can induce robust anti-tumor effect when co-cultured with PBMCs (Fig. 2D, *P* < 0.05). We pretreated cancer cells and PBMCs with a gradient concentration of dexamethasone and identified the most effective concentration as 50 nM (Fig. 2E). And the anti-tumor effect of dexamethasone could be rescued by overexpression of PD-L1 or IDO1 in cancer cells (Fig. 2F and Supplementary Fig. S2D).

Besides dexamethasone, there are several other glucocorticoids widely used in clinic, including betamethasone, hydrocortisone, and prednisolone. Our results indicated that the suppression of these glucocorticoids on PD-L1 and IDO1 signaling pathway is weaker than that of dexamethasone (Supplementary Fig. S3A–S3B). Moreover, we found IDO1 inhibitor (Epacadostat) can increase the expression of IDO1 and PD-L1 in some cancer cell lines (Supplementary Fig. S3C). We further explored the effect of dexamethasone on PD-L1 and IDO1 expression of T cells. Dexamethasone could decrease the viability and PD-L1 expression of CD4+ T cells in PBMCs (Supplementary Fig. S4A–4B), but did not alter IDO1 expression of PBMCs (Supplementary Fig. S4C).

### Dexamethasone increases the efficacy of ICIs in vitro

**Fig. 3:**
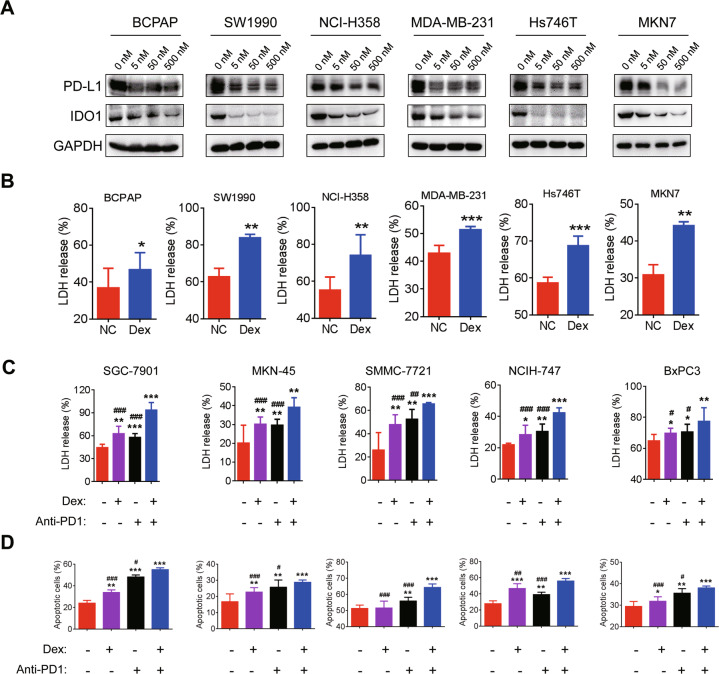
Dexamethasone increases efficacy of ICIs in vitro. **A** After treatment with dexamethasone for 48 h, expression of PD-L1 and IDO1 in BCPAP, SW1990, NCI-H358, MDA-MB-231, Hs746T, and MKN7 cells were detected by Western blot; (**B**) Cancer cells were incubated with dexamethasone for 48 h and then co-cultured with PBMCs with effector/target ratio of 10:1. LDH level was detected; (**C**) Cancer cells were incubated with dexamethasone for 48 h followed by co-cultured with PBMCs with effector/target ratio of 10:1 and the addition of PD1 inhibitor (pembrolizumab; 5 μg/mL) for another 48 h. LDH level was detected. **D** Apoptosis rates were compared in different groups. “*” compared to NC; “#” compared to dexamethasone + anti-PD1. *P < 0.05; **P < 0.01; ***P < 0.001. #*P* < 0.05; ##*P* < 0.01; ###*P* < 0.001.

The above results indicated that dexamethasone can decrease PD-L1 and IDO1 expression and enhance immunosurveillance of T cells. We further validated that dexamethasone can also suppress the PD-L1 and IDO1 pathway in several other cancer cell lines, including thyroid cancer cell line BCPAP, pancreatic cancer cell line SW1990, lung cancer cell line NCI-H358, breast cancer cell line MDA-MB-231, and GC cell lines Hs746T and MKN7 (Fig. 3A). Furthermore, dexamethasone enhanced the anti-tumor effect of T cells (Fig. 3B). To validate the effect of dexamethasone on ICIs, we pretreated responsive cell lines and PBMCs with dexamethasone and PD1 inhibitor (pembrolizumab, 5 μg/mL). The level of LDH and apoptosis were significantly higher in those treated with dexamethasone plus PD1 inhibitor than other groups (Fig. 3C–D).

### Nuclear translocation of GR/STAT3 complex is responsible for the transcriptional suppression of PD-L1 and IDO1

**Fig. 4:**
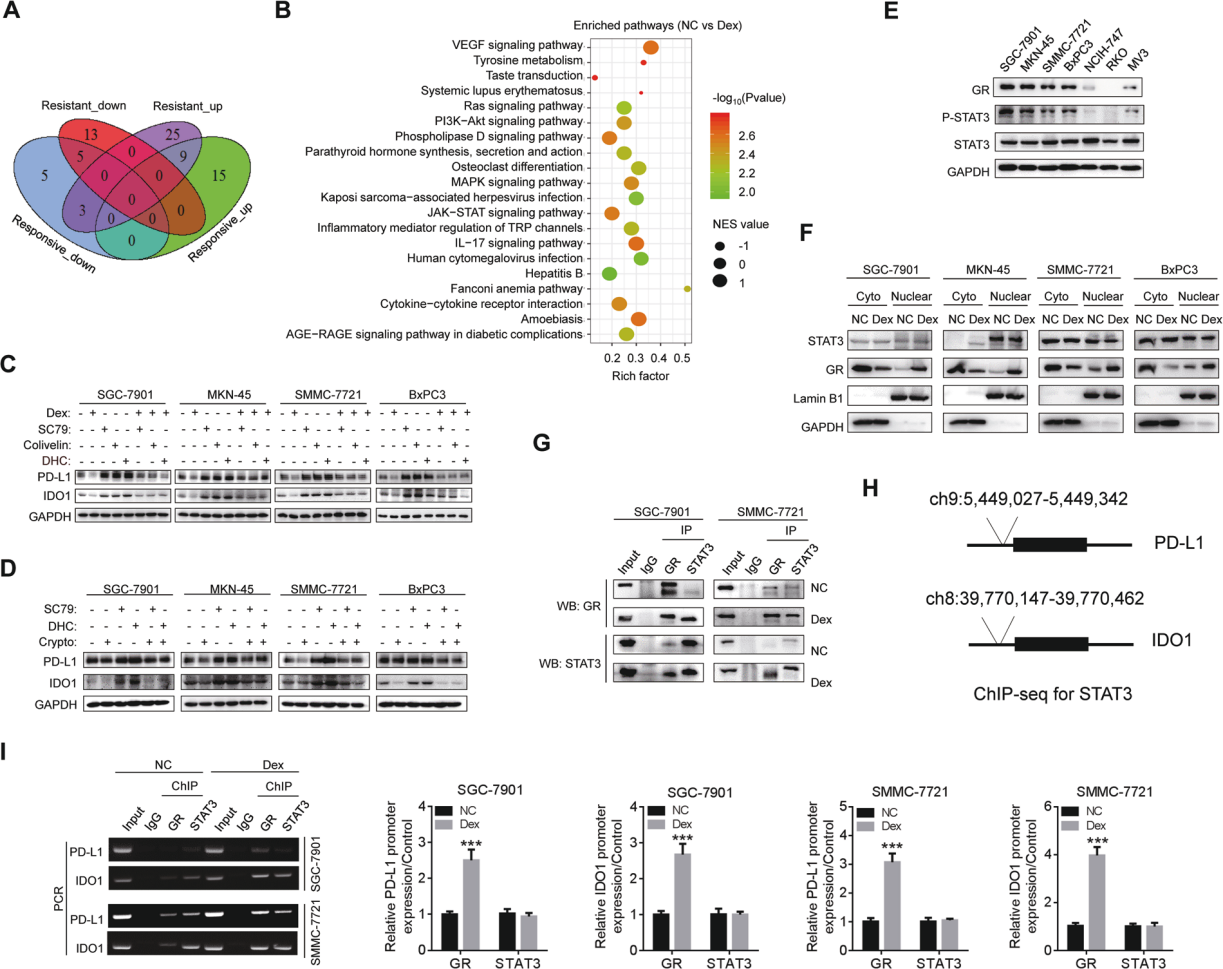
Analysis of pathways and regulatory molecules of dexamethasone on PD-L1 and IDO1 in several types of cancers. **A** Venn diagram to show the enriched pathways in responsive and resistant cells by GSEA analysis (*P* < 0.05; FDR < 0.25). Blue: downregulated pathways of responsive cells. Red: downregulated pathways of resistant cells. Green: upregulated pathways of responsive cells. Purple: upregulated pathways of resistant cells. **B** The top 20 pathways involved in responsive cells; compared to control (*P* < 0.05; FDR < 0.25). Positive and negative NES value indicated the positively and negatively involved pathways by dexamethasone treatment. NES normalized enrichment value, Rich factor: percentage of genes enriched in the pathway. **C** Responsive cells were incubated with PI3K/AKT activator (SC79; 1 μM); MAPK activator (DHC Dehydrocorydaline chloride; 1 μM), JAK/STAT3 activator (Colivelin; 1 μM) or dexamethasone for 48 h, and then protein levels of PD-L1 and IDO1 were detected. **D** AKT activator (SC79; 1 μM) and ERK activator (DHC; 1 μM) mediated upregulation of PD-L1 and IDO1 could be attenuated by STAT3 suppression (Cryptotanshinone; 1 μM). **E** Expression of GR, p-STAT3 and STAT3 in SGC-7901, MKN-45, NCIH-747, RKO, SMMC-7721, BxPC3, and MV3 cells were detected. **F** GR expression in cytoplasm and nucleus of cancer cells was detected after treatment with dexamethasone (50 nM) for 24 h. **G** Co-immunoprecipitation assay to evaluate formation of GR/STAT3 complex in SGC-7901 and SMMC-7721 cells treated with dexamethasone (50 nM) for 48 h. **H** Potential binding sites of STAT3 in the promoters of PD-L1 (ch9:5449027-5449342) and IDO1 (ch8:39770147-39770462) were shown in UCSC ChIP database. **I** ChIP assay to examine the binding of GR and STAT3 on the promoters of PD-L1 and IDO1. IgG served as a negative control.

To explore the mechanisms of dexamethasone-mediated suppression of PD-L1 and IDO1, we stratify the cell lines mentioned above into “responsive cell lines” (SGC-7901, MKN-45, SMMC-7721, and BxPC-3) and “resistant cell lines” (RKO and MV3) according to their response to dexamethasone. NCIH-747 is an intermediate responsive cell line to dexamethasone. Then we performed RNA-seq on these cell lines treated with dexamethasone and identified differentially activated and inactivated pathways (Fig. 4A and Supplementary Table S3). The responsive cell lines showed activated MAPK, PI3K/AKT, and JAK/STAT3 pathways (Fig. 4B). Interestingly, activation of MAPK, PI3K/AKT, and JAK/STAT3 pathways can activate PD-L1 and IDO1 pathways, thus forming a feed forward circuit to enhance immune evasion (Fig. 4C). Furthermore, inhibition of JAK/STAT3 pathway can reverse the upregulation of PD-L1 and IDO1 mediated by activation of MAPK, PI3K/AKT pathway, indicating STAT3 may play critical roles in mediating the function of dexamethasone (Fig. 4D).

Responsive cell lines (SGC-7901, MKN-45, SMMC-7721, and BxPC3) showed higher expression of phosphorylated STAT3 and glucocorticoid receptor (GR) than resistant cell lines (RKO and MV3) (Fig. 4E). We found dexamethasone can increase nuclear translocation of GR but not STAT3 (Fig. 4F). Dexamethasone promoted GR/STAT3 complex formation in nucleus was further validated by Co-IP assay (Fig. 4G). To further identify the mechanism of GR/STAT3 on mediating PD-L1 and IDO1 pathway, we predicted the potential binding sites of STAT3 on the promoter regions of *PD-L1* (ch9:5449027-5449342) and *IDO1* (ch8:39770147-39770462) via UCSC ChIP database (https://genome.ucsc.edu/index.html) (Fig. 4H), and further demonstrated dexamethasone can enhance GR binding to PD-L1 and IDO1 promoter by ChIP assay (Fig. 4I).

### Dexamethasone increases efficacy of ICIs in the ex vivo organoid models

**Fig. 5:**
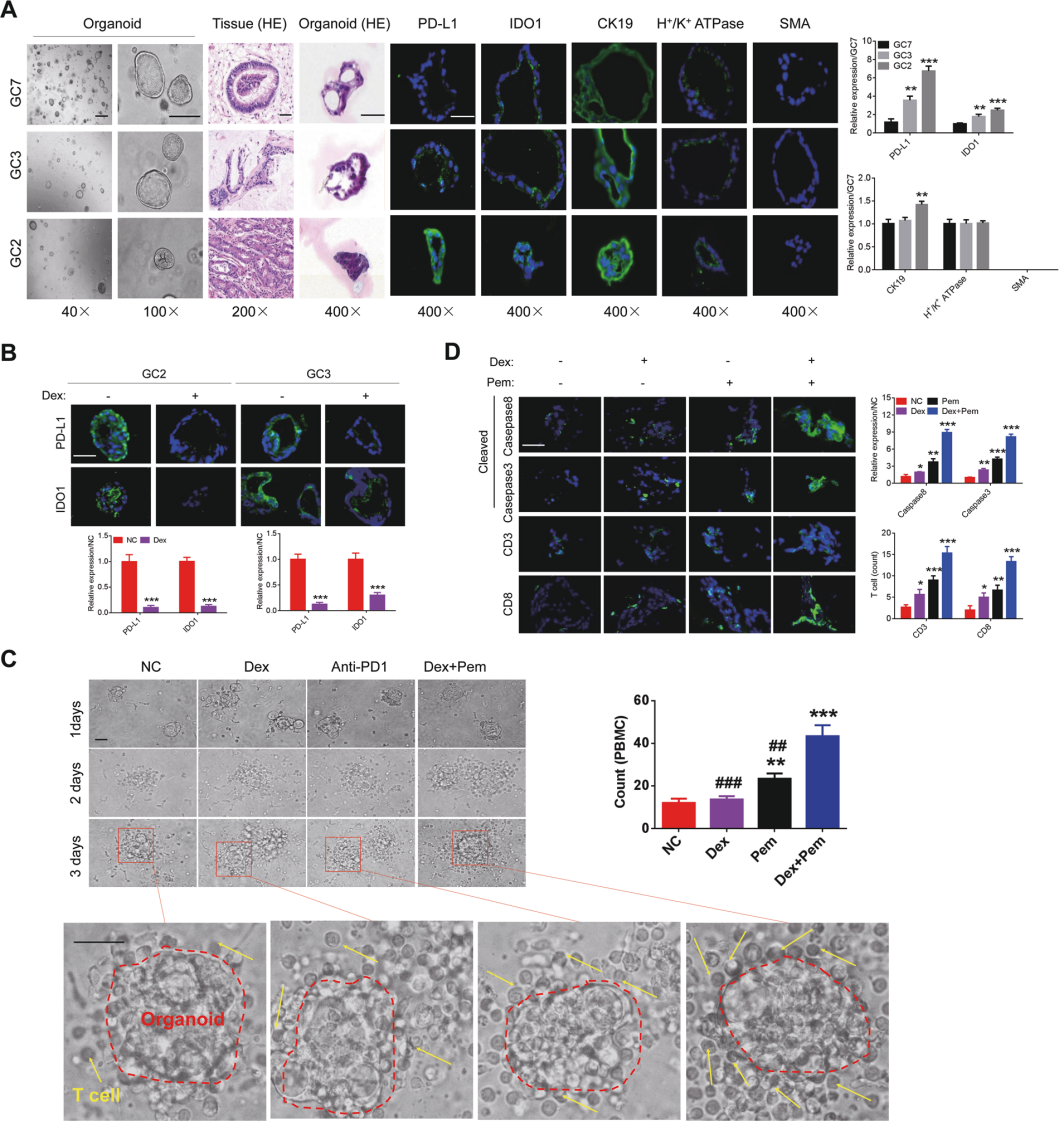
Dexamethasone increases efficacy of ICIs in the ex vivo organoid models. **A** Organoids were constructed from fresh GC tissues (left; 40× and 100×); and HE staining of organoids and organoids-derived tissues was performed (200× and 400×). The expression of PD-L1, IDO1, CK19, H+/K+ ATPase, and SMA on organoids was detected by immunofluorescence. **B** Expression of PD-L1 and IDO1 in organoids (GC3 and GC2) treated with low-dose of dexamethasone (200×). **C** After pretreatment with 50 nM dexamethasone for 72 h, organoids were co-cultivated with PBMCs at an effector/ target ratio of 5:1, and then incubated with dexamethasone (10 nM) or PD1 inhibitor (10 μg/ml) for 48 h. Bright-field microscopy image showed the PBMCs surrounding organoids (200× and 400×); **D** The cells surrounding around organoid were evaluated for the expression of CD3, CD8, caspase3, and caspase8 by immunofluorescence (400×). “*” indicates comparison with NC; “#” indicates comparison with “dexamethasone and pembrolizumab”. The results were repeated three times and shown as the mean ± SD.

We successfully constructed organoids from the above GC tissue with high activity of PD-L1 and IDO1 pathway. Organoids expressed epithelial marker CK19, stomach specific marker H+/K+ ATPase, but not muscular marker SMA (Fig. 5A). Dexamethasone can decrease the activity of PD-L1 and IDO1 pathway (Fig. 5B). Organoids treated with ICIs and dexamethasone recruited more CD3+ and CD8+ T cells and expressed higher levels of cleaved Caspase3 and cleaved Caspase8, indicating enhanced immunosurveillance by T cells (Fig. 5C, D).

### Dexamethasone enhances efficacy of ICIs in vivo

**Fig. 6:**
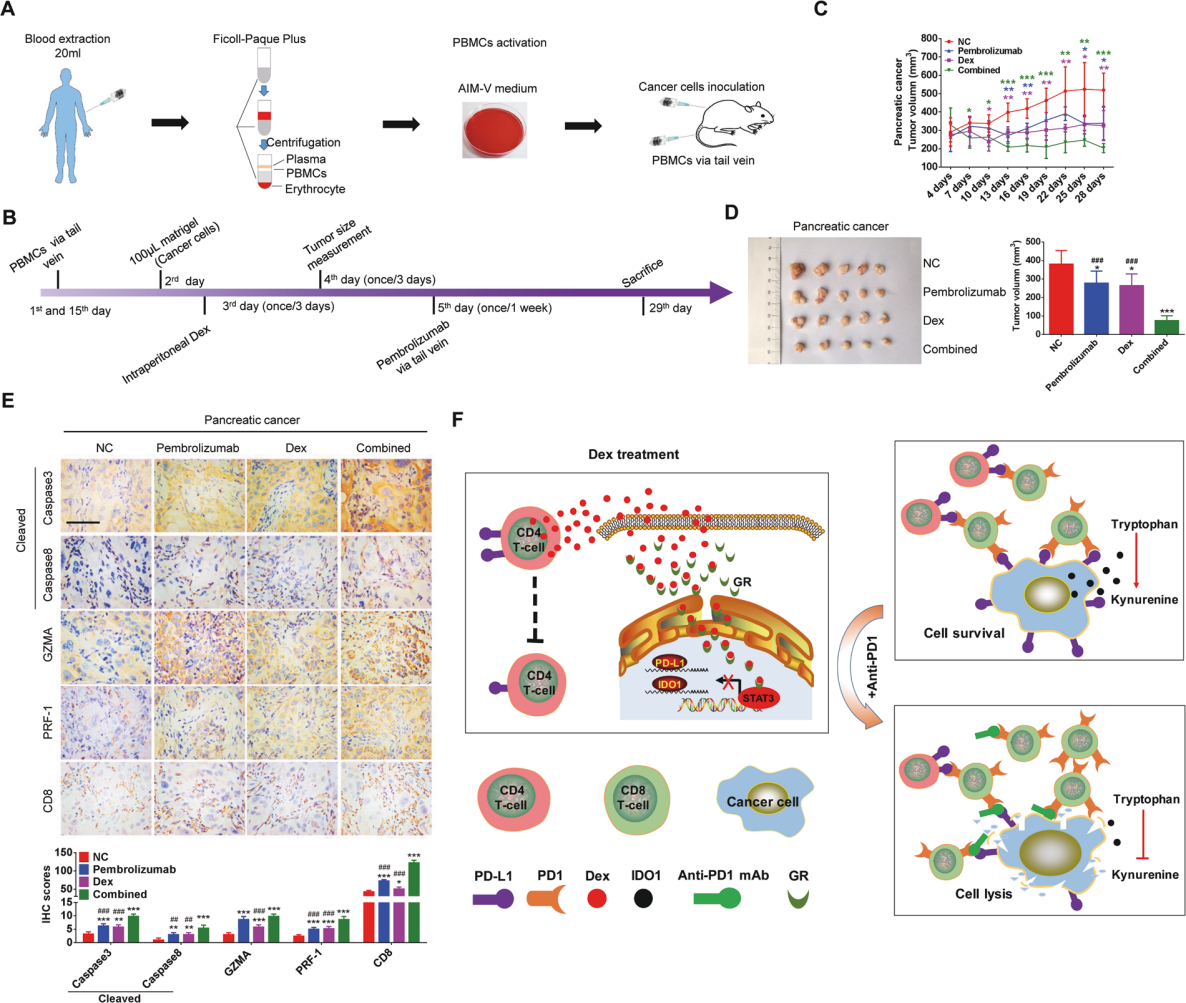
PD1 inhibitor and dexamethasone synergistically suppressed tumor growth of pancreatic cancer in vivo. **A** The schematic flowchart of xenograft model with humanized immune system. **B** The timeline of drug delivery of dexamethasone and anti-PD1 in humanized xenograft model. **C** Tumor growth rates of SW1990 in immune reconstituted model treated by dexamethasone or/and Pembrolizumab. Dexamethasone (0.1 mg/kg) was injected intraperitoneal every 3 days and Pembrolizumab was given via tail vein every week. **D** The sizes of subcutaneous tumor masses removed from the xenograft model. **E** Expression of cleaved caspase3, cleaved caspase8, GZMA, PRF-1, and infiltration of CD8+ cells in mice tissue were examined. (IHC 400×). **F** Schematic diagram of this study. On the one hand, low-dose dexamethasone can inhibit STAT3-mediated PD-L1 and IDO1 transcriptional activity by promoting GR trafficking into the nucleus to form a transcriptional repressor complex GR/STAT3. On the other hand, it can also inhibit expression of PD-L1 in CD4+ T cells, thus reversing tumor immune evasion. “*” compared to NC; “#” compared to Combined group; which indicated “Pembrolizumab + dexamethasone”. Bar; 50 μm. *P < 0.05; **P < 0.01; ***P < 0.001. #*P* < 0.05; ##*P* < 0.01; ###*P* < 0.001.

To verify the efficacy of dexamethasone on enhancing efficacy of ICIs in vivo, we constructed humanized mouse model (Fig. 6A). After the inoculation of cancer cells for 2 days, dexamethasone was injected intraperitoneally every 3 days for 3 weeks. Four weeks after inoculation, all mice were sacrificed and tumor size was measured. Dexamethasone can decrease tumor growth by suppressing PD-L1 and IDO1 pathway, which is consistent to the in vitro and ex vivo findings (Supplementary Fig. S5A–5C). IHC analysis showed that GR was significantly accumulated in the nucleus of cancer cells in groups treated with dexamethasone (Supplementary Fig. S5C). We further explored whether combination of ICIs and dexamethasone can more effectively suppress tumor growth in this humanized mouse model. We reconstructed immune system of NOD/SCID mice by infusing PBMCs (2 × 10^7^) at 1st day and 14th day via tail veins (Fig. 6A). One day after the first PBMCs infusion, GC cell line SGC-7901 and pancreatic cancer cell line SW1990 (5 × 10^6^ cells) were inoculated subcutaneously. Dexamethasone was given intraperitoneally from the 3rd day, and repeated every 3 days (Fig. 6B). At the 5th day, PD1 inhibitor was administrated via tail vein injection, and repeated every 7 days (Fig. 6B). Dexamethasone can enhance the anti-tumor of PD1 inhibitor (Fig. 6C, D). CD8+ cells and Caspase3, Caspase8, GZMA, and PRF-1 pathway were also increased by the addition of dexamethasone (Fig. 6E). To illustrate the critical roles of T cells on mediating the efficacy of dexamethasone, we also conducted the non-humanized mouse model which has severely impaired T cells functions and found that the dexamethasone did not have increased anti-tumor effect in this scenario (Supplementary Fig. S5D–G). Synergistic anti-tumor activity of PD1 inhibitor and dexamethasone was observed in GC (Supplementary Fig. S6A–C). These results indicate that dexamethasone enhanced anti-tumor effect of ICIs is dependent on the activity of T cells, which is regulated by the decreased PD-L1 and IDO1 on cancer cells and PD-L1 on CD4+ T cells (Fig. 6F).

## Discussion

The therapeutic efficacy of immune checkpoint inhibitors has drawn great attentions in the field of cancer research [23]. However, the limited response rates prompt us to explore underlying molecular mechanisms [24]. One strategy is to target multiple immune checkpoints simultaneously [11, 25], but the results varied in different cohorts [26]. It is partially attributed to the limited understanding of the correlation between different immune checkpoints. Currently, there are several publicly accessible databases providing the high-throughput sequencing data [13, 23]. We analyzed correlations of different immune checkpoints in CCLE and TCGA database, and found the close correlation between PD-L1 and IDO1 in several types of cancers. Epacadostat, an inhibitor of IDO1 has been used as adjuvant drug of anti-PD1 in clinical trials [27]. Combination of anti-PD1 and epacadostat showed some efficacy in a phase I/II clinical trial of head and neck squamous cell carcinoma and melanoma [25], but failed in a phase III clinical trial [26]. Currently, the indication of IDO1 inhibitor is unclear. In our study, using IDO1 inhibitor alone could not get an expected result, but resulted in upregulation of IDO1 and PD-L1 in several types of cancer cells.

Drug repositioning is an emerging field. There are several publicly accessible databases in this area [28]. CMap is a database which provides transcriptomic data of nine human cancer cell lines treated with over 2000 compounds, which helps researchers to outline potential compounds that may target specific gene signatures [15, 29]. Using this strategy, we identified a group of compounds that may inhibit PD-L1 and IDO1 simultaneously, among which, dexamethasone is the optimal one. Dexamethasone is one of the glucocorticoids that is widely used as supportive treatment to reduce side effects of chemotherapy [30]. It also has anti-tumor effect in pancreatic and prostate cancer [31, 32]. Calagua et al. reported that prednisone, another glucocorticoid can decrease PD-L1 expression in prostate cancer [33]. Ott et al. found that dexamethasone can decrease the level of tryptophan-2, 3-dioxygenase (TDO), one of rate-limiting enzymes of tryptophan metabolism in human gliomas [34]. Badros et al. found that low-dose dexamethasone at the initial stage of treatment is helpful to improve efficacy of anti-PD1 treatment in multiple myeloma [35]. Arbour et al. indicated that high-dose of glucocorticoid (prednisone, 10 mg/kg) at the initial stage cannot improve the efficacy of immunotherapy [30]. Hence, when using corticosteroids prior to PD-(L)1 blockade, dosage is one of the main factors affecting the efficacy of immunotherapy [36]. In mice, high dose of dexamethasone (1.25 mg/kg) could significantly impair proliferation of mature T cells and diminish the efficacy of anti-PD-1 therapy [37–39]. Regarding to above concerns, we paid a special attention on dexamethasone concentration. We found that low-dose dexamethasone (0.1 mg/kg) not only inhibited the expression of PD-L1 and IDO1 of cancer cell simultaneously, but also reduced the expression of PD-L1 on CD4+ lymphocytes. PD-L1 from both cancer cells and immune cells could attenuate cytotoxic T-cell function and activity in the tumor microenvironment [40]. Our results indicated that low-dose dexamethasone may also enhance anti-tumor immunity by decreasing PD-L1 on surface of CD4+ lymphocytes. Besides, we found that higher dose dexamethasone (0.5 mg/kg and 5 mg/kg) promoted cancer growth. These results indicated that low-dose dexamethasone has anti-tumor activity by suppressing PD-L1 and IDO1, but high-dose dexamethasone induced tumor growth by suppressing tumor immune surveillance, which indicates dexamethasone as a double-edged sword [41].

PD-L1 and IDO1 are regulated by JAK/STAT, MAPK, and PI3K/AKT pathways [42–44], which can also regulate immune escape [45, 46]. Several studies demonstrated that promoters of PD-L1 and IDO1 have binding sites of STAT3 [47, 48]. Dexamethasone regulates downstream genes expression via GR [49]. We confirmed the increased nuclear translocation of GR by dexamethasone treatment, and identified a transcription inhibitory complex of GR/STAT3 playing a role on suppressing PD-L1 and IDO1 expression. To validate the new findings, we further observed therapeutic efficacy of low-dose dexamethasone on enhancing the efficacy of anti-PD1 immunotherapy in fresh cancer organoids and animal models. Organoid is a 3D living system, which is constructed from fresh cancer tissues [50]. Since the gene expression profile of organoids was similar to that of primary cancer tissues [50, 51], the experimental therapy on organoid can mimic the real tumor microenvironment. This study shows that dexamethasone can increase the efficacy of ICIs in cancer organoids and mouse models, thus highlighting the potential of dexamethasone on improving the response of ICIs.

The strengths of this study are: the usage of multiple cancer cell lines, the incorporation of bioinformatic screening and in vitro study and the validation in the ex vivo and in vivo models. This study also has limitations, including the validation of the efficacy of dexamethasone in patients receiving ICIs is lacking and the mechanism of the positive correlation between PD-L1 and IDO1 remains uncharacterized.

In conclusion, we identified dexamethasone as a potent suppressor of tumor immune evasion, which can target PD-L1 and IDO1 simultaneously. Dexamethasone can increase the sensitivity of ICIs in vitro, ex vivo and in vivo by triggering nuclear translocation of GR/STAT3 and transcriptional suppression of PD-L1 and IDO1. This study provides preclinical evidence of dexamethasone as a potential treatment to increase response to ICIs.

## Supplementary information

Supplementary figures

Supplementary Table S1

Supplementary Table S2

Supplementary Table S3

## Data Availability

All data generated or analyzed during this study are included in this published article and the supplementary files.
