# Supplementary material for: Dexamethasone suppresses immune evasion by inducing GR/STAT3 mediated downregulation of PD-L1 and IDO1 pathways
Source: Oncogene. 2021 Jun 26;40(31):5002–12. doi: 10.1038/s41388-021-01897-0 (PMC8235907; doi:10.1038/s41388-021-01897-0)
Supplement: Supplementary file 1 — Supplementary figures [file 41388_2021_1897_MOESM1_ESM.docx]

**Dexamethasone suppresses immune evasion by inducing GR/STAT3 mediated downregulation of PD-L1 and IDO1 pathways**

Zhen Xiang^1*^, Zhijun Zhou^2,3*^, Shuzheng Song^1*^, Jun Li^1^, Jun Ji^1^, Ranlin Yan^1^, Jiexuan Wang^1^, Wei Cai^1^, Wenjun Hu^1^, Lu Zang^1^, Zhenggang Zhu^1^, Zhen Zhang^4#^, Min Li^2,3#^, Yingyan Yu^1#^

^1^ Department of General Surgery of Ruijin Hospital, Shanghai Institute of Digestive Surgery, and Shanghai Key Laboratory for Gastric Neoplasms, Shanghai Jiao Tong University School of Medicine, Shanghai, China.

^2^ Department of Medicine, The University of Oklahoma Health Sciences Center, Oklahoma City, OK 73104;

^3^ Department of Surgery, The University of Oklahoma Health Sciences Center, Oklahoma City, OK 73104;

^4^ Department of Radiation Oncology and Department of Oncology, Shanghai Medical College, Fudan University Shanghai Cancer Center, Shanghai, PR China, 270 Dong An Road, Shanghai, 200032, China.

^*^These authors contributed equally to this work.

**Financial support:** This project is partially supported by the Chinese National Key Program (MOST-2017YFC0908300 and 2016YFC1303200), the National Natural Science Foundation of China (82072602 and 81772505), Shanghai Science and Technology Committee (20DZ2201900 and 18411953100), the cross-institute innovation foundation of Shanghai Jiao Tong University (YG2017ZD01), the Innovation Foundation of Translational Medicine of Shanghai Jiao Tong University School of Medicine (TM202001, 15ZH4001, TM201617 and TM 201702), Technology Transfer Project of Science & Technology Dept. Shanghai Jiao Tong University School of Medicine, and Innovation Foundation for Doctor’s Degree (BXJ201914) grants to Y. Yu.

**# Corresponding authors**:

Yingyan Yu, MD, PhD.

Department of General Surgery of Ruijin Hospital, Shanghai Institute of Digestive Surgery, and Shanghai Key Laboratory for Gastric Neoplasms, Shanghai Jiao Tong University School of Medicine, Shanghai, China. No.197 Ruijin 2nd Road, Shanghai, 200032, China.

Tel: +86-20-34187336, Fax: +86-20-34187336, Email: [yingyan3y@sjtu.edu.cn](mailto:yingyan3y@sjtu.edu.cn)

Min Li, PhD

Department of Medicine, Department of Surgery

The University of Oklahoma Health Sciences Center

975 NE 10th Street, BRC 1262A, Oklahoma City, OK 73104

Tel: (405) 271-1796, Fax: (405) 271-1476, Email: [Min-Li@ouhsc.edu](mailto:Min-Li@ouhsc.edu)

Zhen Zhang, MD

Department of Radiation Oncology and Department of Oncology, Shanghai Medical College, Fudan University Shanghai Cancer Center, Shanghai, PR China

270 Dong An Road, Shanghai, 200032, China.

Tel: +86-21-64175590, Email: zhen_zhang@fudan.edu.cn

**Supplementary Figures.**

**
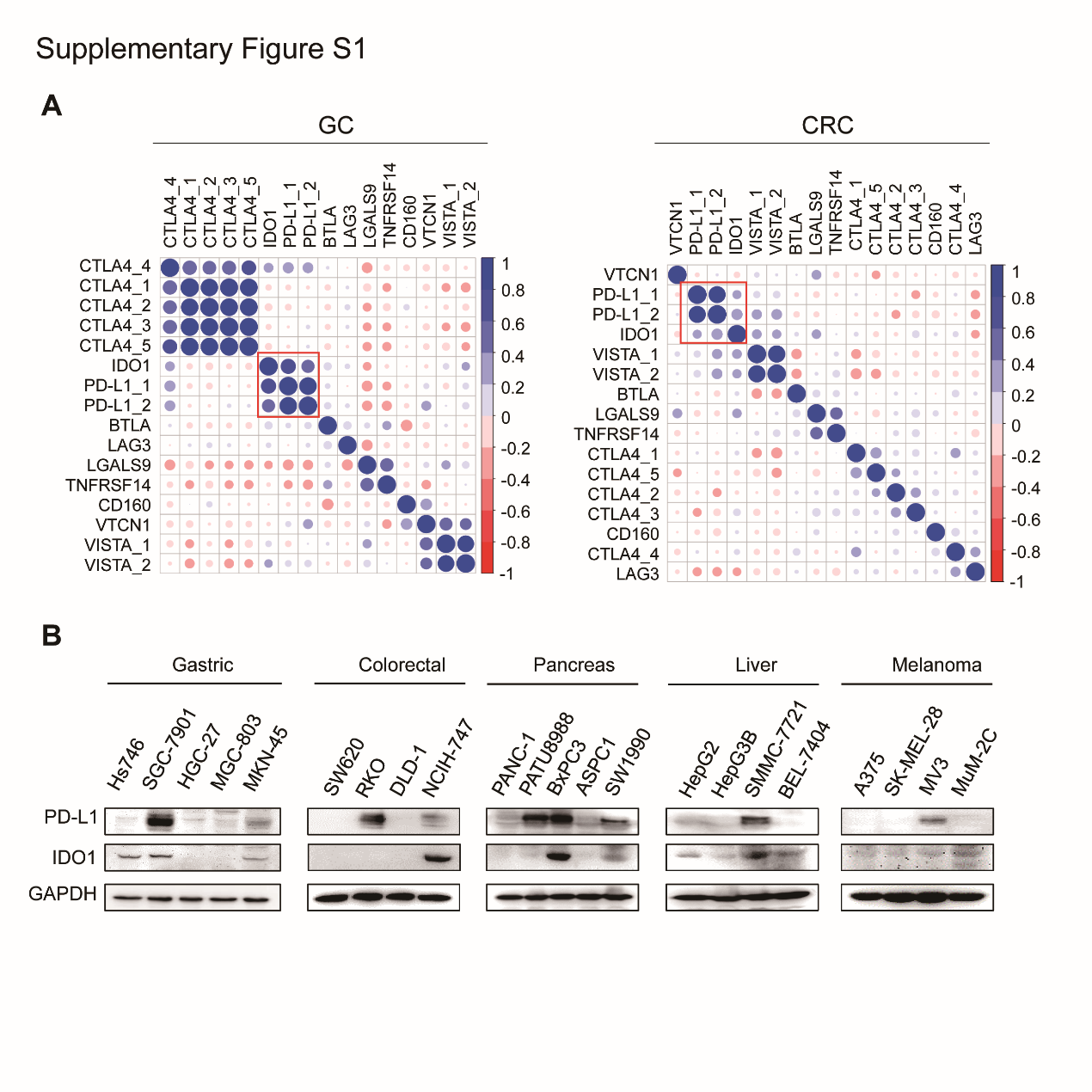
**

**Supplementary Figure S1. Co-expression patterns of multiple immune-related molecules in cancers**. **A,** Correlation coefficients of 10 immune-related molecules in GC and CRC were applied for hierarchical cluster analysis. The red blocks represented negative correlation and the blue blocks represented positive correlation. Larger points indicate stronger correlations. **B,** Expression of PD-L1 and IDO1 in different cancer cell lines by Western blot.
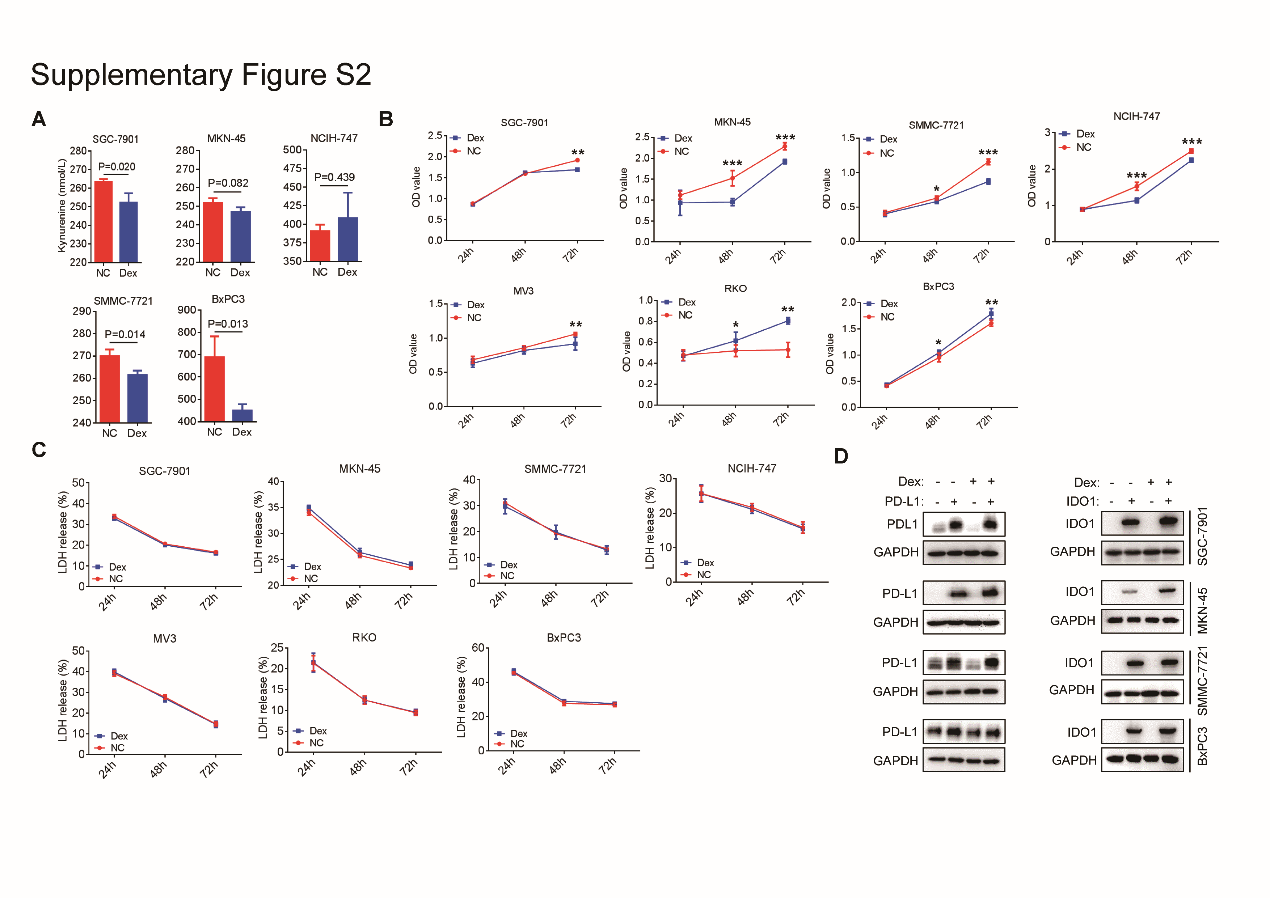


**Supplementary Figure S2**. **The effect of low-dose dexamethasone on cell proliferation and release of kynurenine and LDH.** **A,** Kynurenine levels in cell medium from several types of cancers after 50 nM dexamethasone treatment for 48h. **B,** Cell proliferation assay was examined by CCK8 assay. **C,** After treated with 50nM dexamethasone, LDH was detected. **D,** Cancer cells were treated with 50 nM dexamethasone for 48h, then transfected with PD-L1 or IDO1 plasmid. The expression of PD-L1 and IDO1 were examined 48h later by Western blot. *P<0.05; **P<0.01; ***P<0.001.


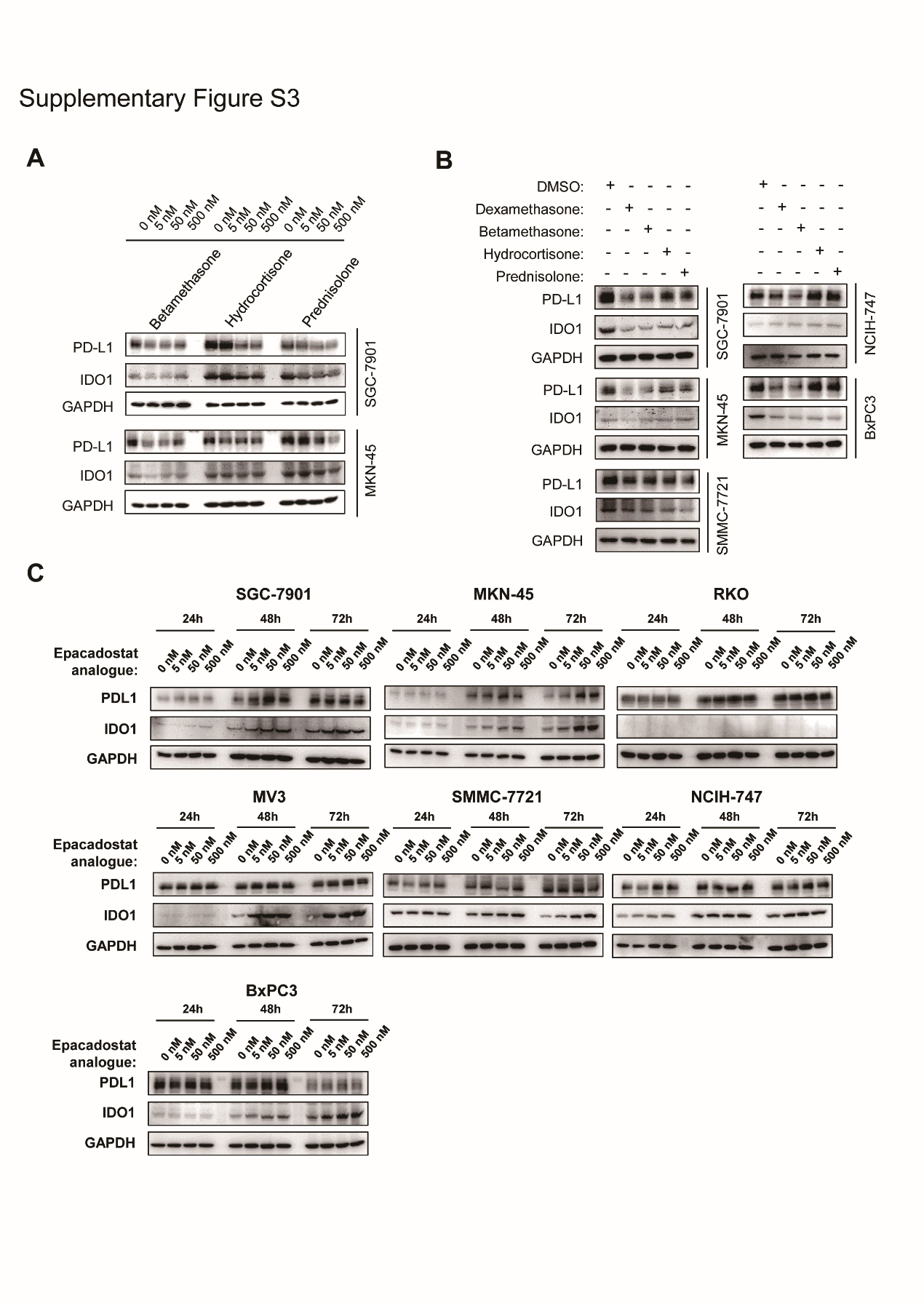


**Supplementary Figure S3**. **Evaluation of several glucocorticoids and IDO1 inhibitor on inhibiting PD-L1 and IDO1 expression.** **A,** After incubation with betamethasone, hydrocortisone and prednisolone on SGC-7901 and MKN-45 cells at different concentrations for 48h, the expression of PD-L1 and IDO1 were detected by Western blot. **B,** After incubation with betamethasone, hydrocortisone and prednisolone of 50nM for 48h in SGC-7901, MKN-45, SMMC-7721, NCIH-747 and BxPC3, the protein expression of PD-L1 and IDO1 were examined by Western blot. **C,** After incubation with epacadostat at different concentrations for 24h, 48h and 72h, the protein expression of PD-L1 and IDO1 were detected by Western blot.


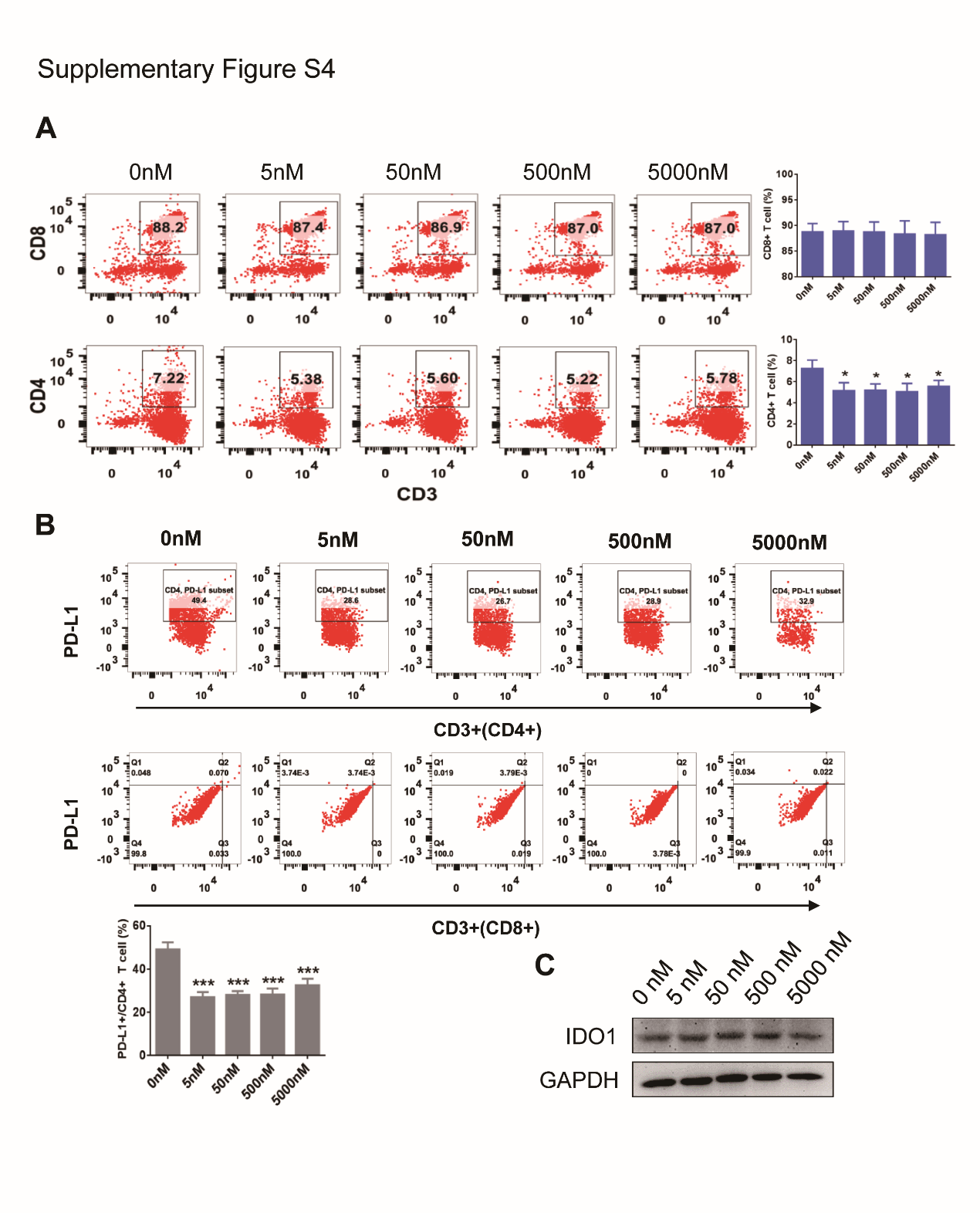


**Supplementary Figure S4**. **The effect of different concentrations of Dex on fractions of T cells from PBMCs, PD-L1 and IDO1 expression in PBMCs.** After incubation of Dex for 48h, the fractions of CD3^+^/CD4^+^ and CD3^+^/CD8^+^ T cells from PBMCs were detected by flow cytometry (A), the expression of PD-L1 on surface of CD4^+^ and CD8^+^ T cells of PBMCs was examined by flow cytometry (B), the expression of IDO1 of PBMCs was detected by Western blot (C).


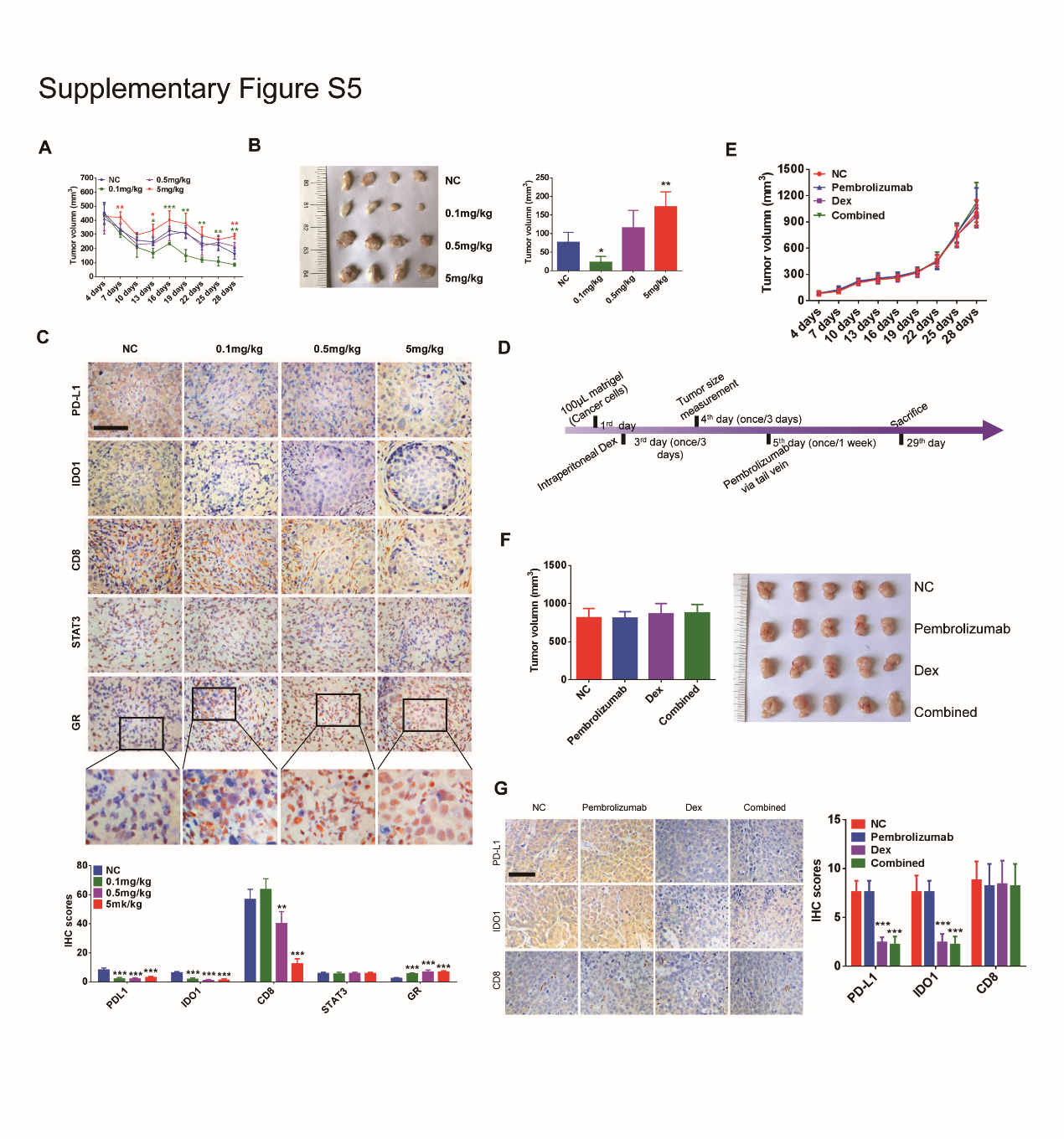


**Supplementary Figure S5**. **Anti-tumor effect of low-dose dexamethasone *in vivo*. A,** Tumor growth rate of SGC-7901 in immune reconstituted model treated with different doses of dexamethasone (0.1mg/kg; 0.5mg/kg; 5mg/kg), which was injected intraperitoneal every three days. The x-axis and the y-axis represent days after injection and tumor size; respectively. “*” indicates comparison with NC group. **B,** The mice were sacrificed 4 weeks after inoculation and tumor volume was measured. **C,** Immunohistochemistry staining was performed to examine expression of PDL1, IDO1 and infiltration of CD8^+^ T cells in tumor tissue (400×). **D,** The timeline of drug delivery of dexamethasone and anti-PD1 in non-immune reconstituted xenograft model. **E,** Tumor growth rate of subcutaneous tumor of SGC-7901 in non-immune reconstituted model treated by dexamethasone or/and Pembrolizumab. Dexamethasone (0.1mg/kg) was injected intraperitoneal every three days. Pembrolizumab was given via tail vein every week. **F,** The sizes of subcutaneous tumor masses removed from the xenograft model. **G,** The expression of PD-L1 and IDO1 in different groups (IHC 400×). *P<0.05; **P<0.01; ***P<0.001. Bar; 50μm.


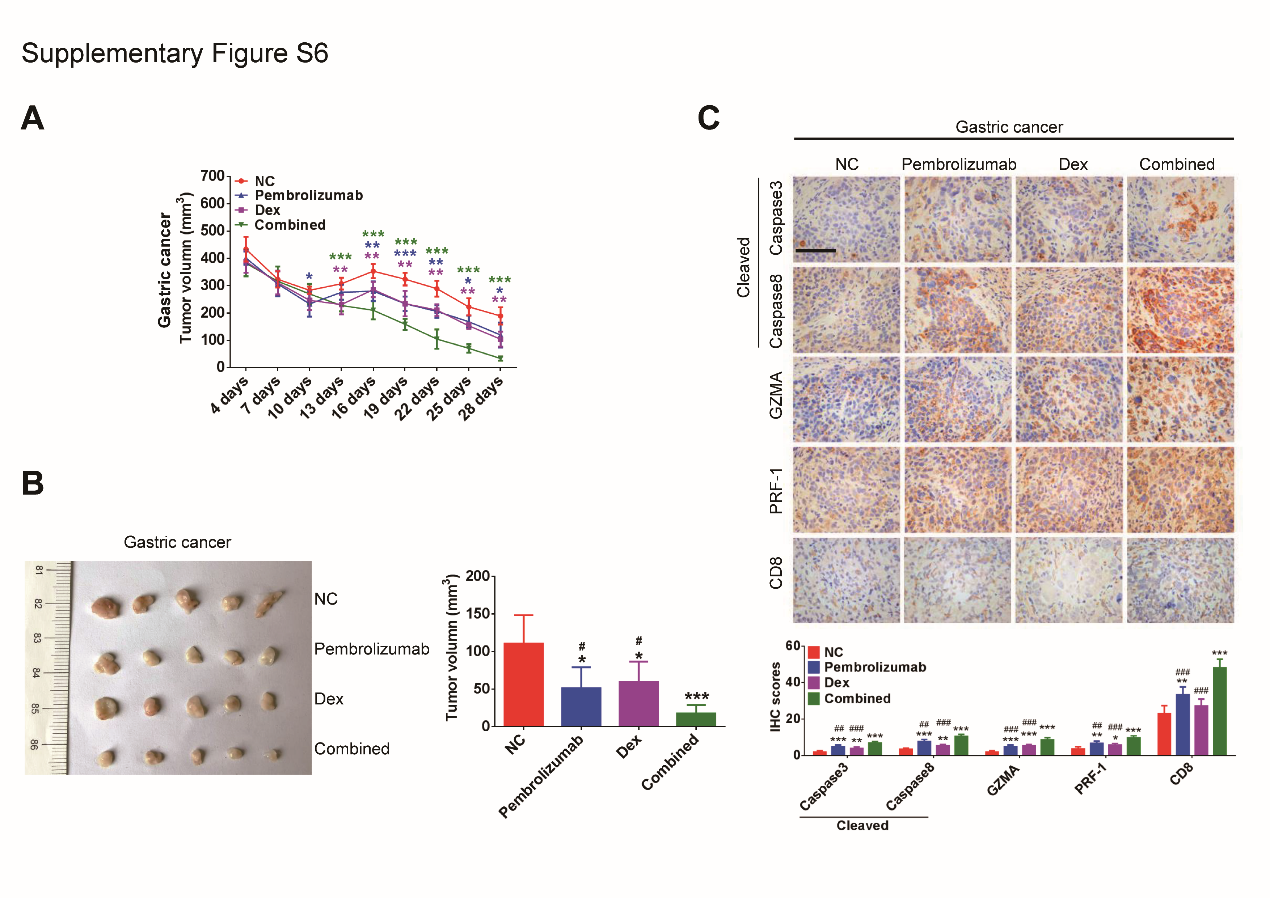


**Supplementary Figure S6. PD1 inhibitor and dexamethasone synergistically suppressed tumor growth of gastric cancer in vivo.** **A,** Tumor growth rates of SGC-7901 in immune reconstituted model treated by dexamethasone or/and Pembrolizumab. Dexamethasone (0.1mg/kg) was injected intraperitoneal every three days and Pembrolizumab was given via tail vein every week. **B**, Tumor sizes of the xenograft models. **C**, Expression of cleaved caspase3, cleaved caspase8, GZMA, PRF-1 and infiltration of CD8+ cells in mice tissue were examined. (IHC 400×). “*” compared to NC; “#” compared to Combined group; which indicated “Pembrolizumab + dexamethasone”. Bar; 50μm. *, P<0.05; **P<0.01; ***P<0.001. #P<0.05; ##P<0.01; ###P<0.001.
